# Cool and hot executive function problems in young children: linking self-regulation processes to emerging clinical symptoms

**DOI:** 10.1007/s00787-023-02344-z

**Published:** 2024-01-06

**Authors:** Kate L. Anning, Kate Langley, Christopher Hobson, Stephanie H. M. van Goozen

**Affiliations:** 1 School of Psychology, Cardiff University, Cardiff, UK; 2 Department of Psychology, Institute of Psychiatry, Psychology & Neuroscience, King’s College London, London, United Kingdom

**Keywords:** Self-regulation, Research Domain Criteria (RDoC), Cognitive control

## Abstract

Self-regulation (SR) difficulties are implicated in a wide range of disorders which develop in childhood, including attention deficit hyperactivity disorder (ADHD), oppositional defiance disorder (ODD), anxiety and depression. However, the integration of the existing research evidence is challenging because of varying terminology and the wide range of tasks used, as well as the heterogeneity and comorbidity within and across diagnostic categories. The current study used the Research Domain Criteria (RDoC) framework to guide the examination of different SR processes in young children showing a wide range of symptomatology. Children (aged 4–8) referred by teachers for moderate-to-high conduct, hyperactivity and/or emotional problems at school (assessed using the Strengths and Difficulties Questionnaire (SDQ) subscales; *n* = 212), and children in SDQ typical ranges (*n* = 30) completed computerised cognitive control and decision-making tasks. Parents completed questionnaires to assess ADHD, ODD, anxiety and depression symptoms (*n* = 191). Compared to children with no teacher-reported difficulties, those with moderate-to-high problems showed poorer visuomotor control and decision-making. A factor analysis revealed that task variables adhered to RDoC dimensions and predicted variance in specific disorders: difficulties in cognitive control predicted ADHD symptoms, low reward-seeking was associated with depression and high reward-seeking was associated with ODD. This study highlights how the assessment of cognitive processes positioned within the RDoC framework can inform our understanding of disorder-specific and transdiagnostic difficulties in SR which are associated with diverse clinical symptoms in children.

**Supplementary Information:**

The online version contains supplementary material available at 10.1007/s00787-023-02344-z.

## Introduction

Self-regulation (SR) refers to an ability to control thoughts, behaviours and emotions in pursuit of goals, despite contrary impulses or distractions [1]. Difficulties in SR are transdiagnostic, playing a role in the development, severity and maintenance of attention deficit hyperactivity disorder (ADHD), emotional (e.g., anxiety, depression) and behavioural (e.g., oppositional defiance disorder; ODD, conduct disorder; CD) disorders [2]. SR problems can be observed from an early age and precede adverse developmental outcomes later in life [3]; thus, SR processes may be useful targets for early intervention [4]. The current study examined how SR processes extracted from cognitive control and decision-making tasks are associated with clinical symptoms in young children identified by teachers as struggling at school.

According to the Research Domain Criteria (RDoC) framework, three domains of functioning are relevant to the study of SR; these are the ‘cognitive’, ‘positive valence’ and ‘negative valence’ systems, which involve controlling attention and inhibition, processing and valuing rewards, and responding to aversive situations such as punishment or loss [5]. Traditional theories of neurodevelopmental disorder (NDD) argued for the existence of disorder-specific cognitive difficulties. For example, classic theories of ADHD highlight the important role of ‘cool’ Executive Functioning (EF), because individuals with ADHD exhibit difficulties on tasks involving working memory, sustained attention and inhibition [6–8]. Indeed, poor regulation of cognitive control under ‘cool’ (non-motivational) conditions is associated with structural and functional differences in the frontal-striatal brain regions of individuals with ADHD [9]. At the same time, processes within the positive/negative valence systems, assessed by tasks which involve regulating responses under ‘hot’ conditions (which include rewards and/or losses), are mediated by orbitofrontal-limbic neural regions [10], and are often compromised in children with ODD/CD. For example, reduced sensitivity to threats of punishment is associated with difficulties in learning to refrain from inappropriate behaviours [11]. In depression, blunted positive affect and anhedonia are associated with altered reward sensitivity [12], resulting in risk aversion [13], whereas individuals with anxiety may struggle to learn from reward-response contingencies due to heightened sensitivity to loss and punishment [14]. Therefore, positioning SR within the RDoC framework and studying processes within each domain (cognitive, positive/negative valence) could offer a consistent terminology to integrate findings and operationalise areas of difficulty which may be associated with problems in regulating cognition, attention, emotions and behaviour in children with emerging clinical symptoms [15, 16].

However, the idea that cool and hot EF processes can differentiate disorders has been challenged by evidence that these difficulties can also co-occur across diagnoses. For example, individuals with ADHD have been found to also show problems with hot EF and altered neural processing of reward and loss, compared to typically developing controls, suggesting that altered reward sensitivity (positive valence) may be a problem to consider in ADHD [17]. Similarly, cool EF problems have been found in ODD/CD [18]; however, these findings are not consistently replicated [19, 20]. In response, recent theories of NDDs acknowledge heterogeneity by incorporating multiple pathways [21]; yet the idea of ‘disorder-specific’ cognitive difficulties persists and influence the design of research studies. Specifically, most research examining cool and hot EF from a psychopathological perspective uses group-level analyses of single processes, comparing those with a clinical diagnosis to typically developing controls. This case–control approach neither captures the high levels of symptom variability nor the varying profiles of strengths and difficulties which exist in those with the same diagnostic label; thus, findings are still confounded by the influences of heterogeneity and the potential for overlapping EF problems between clinical categories [22]. Instead, research examining SR may benefit from adopting a transdiagnostic and broad assessment approach to capture the wide range of EF difficulties and clinical symptoms which are exhibited in those with varying levels of cognitive, emotional and behavioural problems.

A further issue which adds to the complexity of studying SR in children is that ADHD, disruptive behaviour and emotional disorders frequently co-occur. For example, approximately half of children with ADHD meet the criteria for comorbid ODD/CD [23, 24], and almost a third for anxiety [25]. These comorbidities may alter neuropsychological processes [26] but are not always accounted for in studies of cool and hot EF [27, 28]. Therefore, it is currently unclear to what extent disorders, such as ADHD and ODD, have unique or share underlying cognitive difficulties, such as poor inhibition control, and whether comorbidity is associated with co-occurring disorder-specific problems or more severe dysregulation. For example, in children with ADHD and ODD poor cognitive control (unique to ADHD), combined with poor regulation of negative emotions (unique to ODD), may exacerbate impulsive aggression [29]. Conversely, comorbid anxiety in ADHD might counter-act hypoactivation of cognitive control regions and improve cool EF performance [30]. Because the comorbidity rates are so high, using strict exclusion criteria to isolate “pure” cases can greatly reduce the representativeness of a study sample. Comorbidity may also influence cool and hot EF at a sub-threshold level [31]; a dimensional approach that takes co-occurring low-level symptomatology into consideration is, therefore, needed to examine how self-regulatory processes are associated with individual disorder dimensions in children. Furthermore, many children have NDD symptoms without reaching the threshold for a diagnosis and still perform at below age-expected levels on measures of SR processes, such as EF [32]. A dimensional approach to assessment ensures that sub-threshold children who may exhibit SR difficulties and clinical symptoms, and who could benefit from intervention, are not overlooked. In addition, there is a need for studies that use community samples of children identified by teachers as struggling at school [26, 33], rather than those that rely on a clinical diagnosis for inclusion.

The current study utilised a sample of children identified by teachers as having cognitive, emotional or behavioural problems at school to (1) examine to what extent young children exhibit difficulties in cognitive control and decision-making, (2) identify constructs extracted from a range of cognitive control and decision-making task-based measures and (3) examine how these constructs are dimensionally linked to severity of ADHD, ODD, anxiety and depression whilst controlling for co-occurring symptoms. There is limited research which has used a factor analytic approach to collectively examine cognitive, positive and negative valence RDoC processes in primary school-aged children identified by teachers; thus, no strong hypotheses were made [16]. However, we expected to be able to extract constructs that would tap into cognitive, positive and negative valence system functioning and be specifically associated with different clinical symptom dimensions. Specifically, we predicted associations between poorer cognitive control and ADHD severity, better cognitive control and anxiety, higher risk-seeking and ODD, and greater loss sensitivity and depression.

## Method

### Participants

The participants in the study (*n* = 212; boys = 146, girls = 66; mean age = 7, SD = 1.01, age range = 4–8) were selected from a larger sample of children referred to Cardiff University’s Neurodevelopment Assessment Unit (NDAU; https://www.cardiff.ac.uk/neurodevelopment-assessment-unit) by classroom teachers or Special Educational Needs coordinators. Children were included in the current study if they demonstrated moderate-to-high hyperactivity, conduct and/or emotional problems in the school setting, as confirmed by the Strengths and Difficulties Questionnaire (SDQ) categorisation bands (scoring in the top 20% of the population; [34]).

Children who did not have elevated teacher-reported problems (scoring in the ‘close to average’ range), despite being referred by teachers, were used as a comparison group (*n* = 30; boys = 16, girls = 14; mean age = 7, SD = 0.97, age range = 4–8), but were not included in factor structure/dimensional analyses.

According to the Lucid Ability Test [35], children in both groups had a mean estimated general cognitive ability of >70. Children had normal or corrected vision and hearing. No children had a diagnosis of autism spectrum disorder or learning disabilities.

### Background information

Parents provided child and family background information by completing questionnaires, which included details such as ethnic background. Children in the study sample were 87% White British (13% other race/ethnicity; including 3% Asian/Asian British, 9% multiple ethnic groups and 1% not specified).

### Clinical symptoms

#### Strengths and Difficulties Questionnaire (SDQ)

The teacher SDQ [34], completed when making a referral to the NDAU, was used as an inclusion criterion to identify children with moderate-to-high cognitive, emotional and behavioural problems, according to the hyperactivity, emotional and conduct problems scales [36]. The SDQ is a valid and reliable screening tool for assessing the prevalence of childhood mental health difficulties (Cronbach’s alpha 0.73, test–retest reliability 0.62; [37]).

#### Child behaviour checklist (CBCL)—parent version

The CBCL [38] was completed by children’s parents/carers (usually mother). The CBCL is a widely used and well-validated measure of clinical symptoms [39]. For all dimensional analyses (correlations, regression), we used the attention problems, ODD, anxiety, and depression *T* scores. Some children’s parents were unable to complete the CBCL because of time constraints (*n* = 21); thus, the sample of children included in all dimensional regression analyses was smaller than the original sample (*n* = 191). The subscales of the CBCL have demonstrated good validity (Cronbach’s alphas of 0.75–0.85) and test–retest reliability (0.78–0.89) [39].

### Self-regulation processes

#### Cognitive control

Cognitive control was measured using two assessments taken from the Amsterdam Neuropsychological Tasks (ANT) battery [40]. Reliability scores have been calculated for subsets of the tasks (test–retest reliability ranges between 0.70 and 0.85; [41]), and many studies have provided evidence for the construct and discriminant validity of the ANT [42–48]. *Z* scores were used to indicate performance, which are scores that are converted from raw test scores using a nonlinear regression function derived from data of 2,340 typical controls [49]. The ANT-Pursuit and ANT-ROO were used as assessments of cognitive control.

The ANT-Pursuit task requires participants to follow a randomly moving star around a screen for 1 min using a mouse cursor to measure executive control of motor movements and attention [50]. Accuracy is measured as the mean distance between the cursor and the target (mm) over the 1 min duration.

The ANT-Response Organisation Objects (ANT-ROO) test is a measure of response inhibition. In Part 1 of the task, participants have to click on a mouse button, which corresponds to the side on which a red dot is randomly presented. In Part 2, participants are required to click the button on the opposite side to where a white dot is presented, which involves overriding the prepotent response learnt in Part 1. The response time and number of errors on Part 2 are converted to age-standardised *Z* scores by ANT software, and are averaged to capture overall performance.

### Positive/negative valence systems: reward-seeking, sensitivity to loss, decision-making

The hot EF tasks used in the current study are adapted versions of well-established assessments of decision-making under risk; the Balloon Analogue Risk Task (BART) [51] and IOWA gambling task [52]. Previous research has validated these hot EF tasks in two ways. First, the performance of older children, adolescents and adults corresponds with real-life risky behaviour, such as substance use, gambling and criminal behaviour [53, 54]. Second, the tasks elicit autonomic responses following rewards and losses, indicating activation of positive and negative valence processes (e.g., increases in heart rate and skin conductance) [55].

#### Balloon Emotional Learning Task (BELT)

The BELT is a computerised risky decision-making task in which participants pump up balloons and earn points [56]. There are two buttons: one to pump up the balloon and earn points, and one to stop pumping and cash in points. If the balloon explodes, the participant gains zero points for that balloon. Balloons have 3 different colours, which are evenly presented over the task (i.e., there are 9 trials of each colour); pink balloons always explode at 7 pumps (“certain-short”), orange balloons explode at 19 pumps (“certain-long”) and blue balloons explode variably at 7, 13 or 19 pumps (“uncertain”). There are 27 trials. Different sounds are played following a gain of points (a dinging fairground noise) and an explosion sounds when a balloon is popped. We focussed on reward-seeking under certain-short conditions, where feedback is most likely to be encountered [57]. Risk-taking was measured as the proportion of pumps made (number of pumps made/total number of possible pumps), whilst successful decision-making was measured using the proportion of points earnt. We assessed loss sensitivity using post-explosion pump reduction (mean number of pumps - mean number of pumps following an explosion). Positive values indicate fewer pumps on the subsequent balloon (greater loss sensitivity). No studies have looked at the reliability of the BELT. However, this task is based on the Balloon Analogue Risk Task (BART), which shows good internal consistency (*r* = 0.70) and moderate test–retest reliability (0.69; [54, 58]).

#### Hungry Donkey

The Hungry Donkey task is a child version of the Iowa Gambling Task (IGT) [52]. Participants are told to collect apples for the Hungry Donkey by choosing between two doors which are displayed on a computer screen, with an image of a donkey underneath. The net score is presented on the left. Participants are told to collect apples for the donkey by selecting the best door. One door is associated with low gains and low losses, which is advantageous over time (resulting in a net gain). The other door has high gains and high losses (resulting in a net loss). A button box is used to select the left or right door, which reveals green (gained) or red (lost) apples. A chomping noise is played when apples are gained and an aversive error sound is played when apples are lost. There are 50 trials in total. The first ten trials are excluded because risk/loss contingencies have not yet been experienced and learnt [59]. Risk-taking was measured using the total number of disadvantageous choices made under risky conditions, successful decision-making was the net score over the full task and sensitivity to loss was the number of switches to a disadvantageous choice after a loss [60, 61]. Previous studies indicate that the psychometric properties of the IGT and Hungry Donkey task are adequate in terms of internal consistency (0.63–0.69) and construct validity, but weaker in terms of test–retest reliability (0.26–0.27; [58, 62]).

### Verbal ability

#### LUCID

To control for variation in verbal ability, we used age-standardised verbal reasoning scores from the Lucid Ability Computerised Assessment System (Version 5.15) [63]. Two different tasks are administered depending on the child’s age. Children aged 4 to 6 complete the “Picture Vocabulary Test” in which they are asked to select one out of five pictures to match a word. Children over 6 are given the “Link Word” task, in which they have to select a word out of six options which “best” links two pictures together (e.g., “summer” links a picture of sandals and an ice cream). The verbal ability tasks in the LUCID have good test–retest reliability (0.87), validity (Cronbach’s alpha = 0.96), and are comparable to other established verbal ability assessments, such as the Wechsler Intelligence Scale for Children (WISC-III), and the British Picture Vocabulary Scale (Second Edition) [35, 63, 64].

### Procedure

Following a referral from school, children and their primary caregivers (usually mother) visited the NDAU for two 3-h sessions of assessment. Children completed measures of cognitive control and decision-making with a trained researcher, whilst their caregivers completed a clinical interview and questionnaires on family background and child symptomatology in a separate room. The cognitive assessments in the current study which were administered in the first session were delivered in the following order: verbal ability (LUCID), response inhibition (ANT-ROO), BELT. In the second session, 1 week later, participants completed the visuomotor task (ANT-Pursuit) and the Hungry Donkey task. These assessments were interspersed with other tasks to measure other domains of functioning (e.g., theory of mind, emotion recognition; see NDAU website for more information; https://www.cardiff.ac.uk/neurodevelopment-assessment-unit/refer-a-child/our-assessments). Participants received breaks if they felt tired or were restless. The researchers conducting the child assessments and parent interviews did not see the teacher SDQ prior to the session.

### Ethical considerations

We gained informed consent from the caregiver and verbal assent for each child before the assessment and all procedures were approved by the Cardiff University ethics committee (EC.16.10.11.4592GR).

## Data analysis

### Preliminary analysis

We examined associations between SR processes and symptom severity scores with potential confounding variables (age, sex, verbal ability) and checked assumptions (normality, multicollinearity) [65].

### Cognitive control and decision-making difficulties in children with moderate-to-high teacher-reported problems

We compared our sample of children with moderate-to-high teacher-reported problems (*n* = 212) to children with scores in the typical range (*n* = 30) using independent samples *t* tests. We also estimated the prevalence of below-average cognitive control by comparing participant performance to age-equivalent norms [49]. For the decision-making tasks, there are limited studies which have examined performance on the BELT and Hungry Donkey in children of this age group [66, 67]. Therefore, to examine poor performance, we looked at the proportion of children who failed to show improvement as each of the decision-making tasks progressed [60, 61].

### Extracting constructs from a range of cognitive control and decision-making measures

In line with previous studies, a Principal Components Analysis (PCA) with Varimax rotation was used to create constructs from the different tasks [16]. All moderate-to-high risk children were included in this analysis (*n* = 212). All variables were converted to *Z* scores and cognitive control measures (scores extracted from the ANT tasks) were reverse scored so that higher scores reflected better performance. In total, eight variables (visuomotor control, inhibition, 2 × risk-taking (BELT, Hungry Donkey), 2 × loss sensitivity (BELT, Hungry Donkey), % points on the BELT, net score on the Hungry Donkey) were entered into the factor analysis. Between-task bivariate Pearson correlations, the Kaiser–Meyer–Olkin (KMO) measure of sampling adequacy and Bartlett’s test of sphericity were used to determine the appropriateness of a PCA. We extracted regression factor scores from the PCA to create composite scores for each formed construct.

### Associations between extracted constructs and dimensional measures of clinical symptoms

Bivariate Pearson correlations were used to examine associations between the composite scores extracted from the PCA and the CBCL symptom scores in children whose parents completed this questionnaire (*n* = 191). These children were included in all dimensional analyses (correlations and regression models). Where we observed significant correlational associations, linear regression analyses were conducted to examine these relationships further. We entered potential confounding variables (age, sex, verbal ability) in Step 1, co-occurring symptoms (ADHD, ODD, anxiety, depression) in Step 2 and extracted composite scores in Step 3 to examine whether the formed constructs predicted significant variance in specific symptom dimensions.

## Results

### Sample characteristics

To understand the symptoms exhibited in our sample, we looked at the proportion of children scoring in the clinically significant range of the CBCL [38]. These prevalence estimates are purely for illustrative purposes and did not inform subsequent data analyses.

Over half of the sample had scores within the clinically significant range on the CBCL for at least one disorder (*n* = 112, 53%). ADHD symptoms were most prevalent (*n* = 87, 46%), whilst approximately a third of children scored in the clinically significant range for ODD (*n* = 63, 33%), anxiety (*n* = 60, 31%) and depression (*n* = 60, 30%).

### Preliminary analyses

We examined whether children who had missing CBCL data (*n* = 21) differed from children with a full set of data (*n* = 191) on potentially confounding variables (age, sex, verbal ability) and teacher-reported problems, and found no significant differences.

All data followed a normal distribution except for visuomotor control, so we examined Spearman’s correlations to confirm associations where this variable was concerned. Examination of correlations and residual plots in SPSS showed that the assumptions for multiple regression were met.

Age was associated with scoring more points on the BELT (*r* = 0.17, *p* = 0.01). Corrected *t *tests showed that girls showed worse inhibition than boys (*t*(84.21)^1^ = −2.79, *p* = 0.01, *d* = −0.51). Verbal ability was associated with better inhibition (*r* = −0.15, *p* = 0.04).

### Cognitive control and decision-making difficulties in children with moderate-to-high teacher-reported problems

We compared children with moderate-to-high teacher-reported problems (*n* = 212) to a comparison group with no problems (*n* = 30) on measures of cognitive control and decision-making (see Table 1). The groups did not differ in age or verbal ability, but the moderate-to-high risk group had more boys (69% compared to 53%). Independent samples *t *tests showed that children with moderate-to-high problems showed poorer performance on assessments of visuomotor control and decision-making than children with no problems, reflected in worse accuracy on the ANT-Pursuit; *t*(60.20)^1^ = −2.69, *p* = 0.01, *d* = −0.34), and fewer points earnt on the BELT task (*t*(240) = 2.89, *p* < 0. 01, *d* = 0.56).

**Table 1:** Performance of sample on EF measures

	Controls (*n* = 30)	Moderate-to-high risk children (*n* = 212)
*Visuomotor control and attention*		
Mean (SD)	0.80 (1.99)	1.98 (3.59)
Prevalence of children scoring in below-average range: *n* (%)	10 (33)	104 (49)
*Inhibition*		
Mean (SD)	0.45 (1.31)	0.53 (1.32)
Prevalence of children scoring in below-average range: *n* (%)	9 (30)	55 (26)
*Decision-making: BELT*		
Proportion of points earnt		
Mean (SD)	0.47 (0.19)	0.37 (0.18)
Learning failure: *n* (%)	4 (13)	51 (24)
*Decision-making: Hungry Donkey*		
Net score		
Mean (SD)	12.00 (12.50)	12.10 (21.16)
Learning failure: *n* (%)	8 (27)	58 (27)

*BELT* Balloon Emotional Learning Task

As shown in Table 1, in the group of children with moderate-to-high problems, 49% showed poor visuomotor control and attention, and 26% showed below-average inhibition compared to norm data for typically developing children [49]. Approximately a quarter of the sample of children showed no learning of choice-outcome contingencies on the BELT and Hungry Donkey decision-making tasks (24 and 27%, respectively), as measured using improvement between the first and last task blocks [55, 61].

### Extracting constructs from a range of cognitive control and decision-making measures

Our correlational analysis (see Table 2) showed that there were significant correlations across tasks, indicating the presence of shared underlying processes. The KMO and Bartlett test validated that structure detection was appropriate for our data [KMO = 0.545, all individual values were above 0.5; *χ*^2^(28) = 196.44, *p* < 0.001]. The Principal Components Analysis suggested a four-factor solution based on visual inspection of the scree plot; the four components had eigenvalues >1, the factors were interpretable and every variable presented a high loading on one component only [68]. The component loadings for our eight measures are shown in Table 3. Together, the four components formed accounted for approximately 68% of the variance (Table 3). The interpretation of the components was guided by constructs within the positive valence, cognitive systems and negative valence domains of the RDoC framework [5, 16], and previous studies which have shown that some constructs are at the intersection of multiple domains (e.g., impulsivity) [69]. The first component, explaining 21% of the variance, was associated with processes within the “positive valence” system and reflected low reward-seeking as assessed via performance on the Hungry Donkey task (fewer risky choices, more points). The highest loadings on the second component, explaining 18% of the variance, reflected cognitive control processes within the “cognitive systems” domain, with high loadings from measures of inhibition and visuomotor control. The third factor (16% variance explained) was associated with both positive and negative valence; elevated reward-seeking and greater loss sensitivity on the BELT, thus tapping into ‘emotional impulsivity’. The final factor (14% variance explained) was specifically associated with “negative valence”; sensitivity to loss (i.e., more choice switches after a loss on Hungry Donkey task), as well as success rate on the BELT (more points gained). We extracted regression factor scores from the PCA to create individual composite scores for each formed RDoC construct.

**Table 2:** Associations between age, verbal ability, symptom score variables and all task variables in at-risk children

	1	2	3	4	5	6	7	8	9	10	11	12	13
1. Age													
2. Verbal ability	−0.13												
3. ADHD	0.08	−0.04											
4. Anxiety	0.04	−0.07	0.26**										
5. Depression	−0.02	−0.03	−.35**	0.61**									
6. ODD	−0.03	−0.05	0.53**	0.38**	0.54**								
7. Visuomotor control	−0.10	−0.02	0.12	−0.21**	−0.11	−0.08							
8. Inhibition	−0.07	−0.15*	0.19**	−0.05	0.01	0.04	0.42**						
9. Risk-taking (BELT)	0.06	−0.12	0.06	0.08	0.10	0.19**	0.01	0.03					
10. Loss sensitivity (BELT)	0.00	−0.11	−0.07	−0.06	−0.07	−0.14*	0.03	0.14*	0.18**				
11. % Points (BELT)	0.17*	−0.04	−0.12	−0.06	−0.09	−0.06	−0.16*	−0.05	−0.13	−0.05			
12. Risk-taking (HD)	−0.09	−0.09	0.02	−0.11	−0.09	0.10	0.18**	0.17*	0.25**	0.11	−0.19**		
13. Loss sensitivity (HD)	−0.07	−0.10	−0.17*	−0.05	−0.16*	−0.15*	−0.13	0.03	0.01	0.07	0.04	0.16*	
14. Net Score (HD)	−0.07	0.10	0.00	0.11	0.22**	0.04	−0.15*	−0.13	−0.17*	0.01	0.04	−0.59**	0.00

*ADHD* attention deficit hyperactivity disorder, *ODD* oppositional defiance disorder, *BELT* Balloon Emotional Learning Task, *HD* Hungry Donkey

*p < 0.05; **p < 0 .01

**Table 3:** Principal Component Analysis to identify RDoC constructs in our sample using cognitive control and decision-making tasks

	Component			
Construct	Low reward-seeking	High cognitive control	High emotional impulsivity	High loss sensitivity
RDoC domain	(+valence)	(cognitive systems)	(+and −valence)	(−valence)
Visuomotor/attention	0.138	**0.794**	0.052	0.268
Inhibition	0.075	**0.841**	−0.113	−0.107
Risk-taking (BELT)	−0.335	0.175	**0.625**	−0.228
Loss sensitivity (BELT)	0.132	−0.189	**0.788**	0.183
% Points (BELT)	0.145	0.065	−0.365	**0.578**
Risk-taking (HD)	−**0.851**	−0.136	0.191	0.054
Loss sensitivity (HD)	-.155	.058	.217	**.774**
Net score (HD)	**0.871**	0.092	0.089	0.008
Explained variance (%)	21.022	17.984	15.632	13.807
Eigenvalue	2.004	1.301	1.115	1.056

Bold indicates factor loadings >0.40

*BELT* Balloon Emotional Learning Task, *HD* Hungry Donkey

### Associations between extracted constructs and dimensional measures of clinical symptoms

Our correlational analysis examined associations between extracted constructs and CBCL dimensional symptom scores (*n* = 191). As shown in Table 4, the severity of ADHD symptoms was significantly associated with poorer cognitive control, severity of anxiety was significantly associated with better cognitive control and severity of depression was significantly associated with lower reward-seeking. ADHD, ODD and depression symptoms were all significantly negatively associated with “negative valence”.

**Table 4:** Correlational associations between disorder symptom dimensions and formed RDoC constructs

	1	2	3	4
1. ADHD				
2. Anxiety	0.26**			
3. Depression	0.35**	0.61**		
4. ODD	0.53**	0.38**	0.54**	
5. Low reward-seeking (+valence)	−0.01	0.09	0.15*	−0.07
6. High cognitive control (cognitive systems)	−0.16*	0.17*	0.08	0.07
7. High emotional impulsivity (± valence)	-.01	.04	.06	.02
8. High loss sensitivity (−valence)	−0.20**	−0.07	−0.18*	−0.17*

*ADHD* attention deficit hyperactivity disorder, *ODD* oppositional defiance disorder

*p < 0.05; **p < 0.01

Regression analyses were used to examine specific associations between different SR processes and symptom dimensions whilst controlling for age, sex and verbal ability as well as co-occurring clinical symptoms (Table 5, for detailed regression models see Supplementary Material). This demonstrated that ADHD symptoms were specifically associated with poor cognitive control, ODD with greater reward-seeking and depression severity with low reward-seeking. Anxiety was associated with better cognitive control.

**Table 5:** Summary of final step multiple regression analyses examining formed RDoC constructs as predictors of disorder symptom severity, controlling for co-occurring symptoms age, sex and verbal ability

Formed RDoC constructs	Statistic	Symptom dimensions			
		ADHD	Anxiety	Depression	ODD
Step 3:	∆*R*^2^	0.05**	0.02	0.02	0.02
Low reward-seeking	*β*	0.016	0.02	0.13*	−0.13*
(+valence)	95% CI	[−1.13, 1.45]	[−1.13, 1.63]	[0.20, 2.54]	[−2.23, −0.18]
High cognitive control	*β*	−0.20**	0.13*	−0.01	0.10
(cognitive systems)	95% CI	[−3.28, −0.73]	[0.06, 2.83]	[−1.27. 1.15]	[−0.26, 1.83]
High emotional impulsivity	*β*	−0.02	0.01	0.02	0.01
(±valence)	95% CI	[−1.50, 1.03]	[−1.27, 1.43]	[−0.92, 1.41]	[−0.92, 1.12]
High loss sensitivity	*β*	−0.12	0.04	−0.08	−0.01
(−valence)	95% CI	[−2.54, 0.03]	[−0.90, 1.88]	[−2.08, 0.30]	[−1.10, 0.99]

*ADHD* attention deficit hyperactivity disorder, *ODD* oppositional defiance disorder

*p < 0.05; **p < 0.01

## Discussion

Young children exhibiting moderate-to-high cognitive, emotional and behavioural problems at school had difficulties on computerised assessments of visuomotor control and decision-making. An analysis of the underlying structure of the variables extracted from these tasks revealed a four-factor solution, separating the domains of cognitive control, reward-seeking behaviour (positive valance), loss sensitivity (negative valence) and emotional impulsivity (positive and negative valence). We found that problems with cognitive control were associated with severity of ADHD symptoms, and that reward-seeking behaviour (positive valence) was associated with both depression (lower reward-seeking) and ODD (greater reward-seeking). Although greater loss sensitivity (negative valence) was significantly negatively correlated with ADHD, depression and ODD symptoms, these associations were not maintained when age, sex, verbal ability and co-occurring symptoms were controlled for. These results indicate that positioning self-regulation within the RDoC framework and adopting a dimensional approach to clinical assessment may be useful in identifying specific processes that could be targeted for intervention in young children with a range of clinical symptoms.

Consistent with previous research highlighting the multidimensional nature of SR [70, 71], we identified four distinct constructs which corresponded to different self-regulatory processes. Our measures of cognitive control (visuomotor control and attention, response inhibition) mapped onto a single factor and were separate from the variables extracted from hot EF tasks. In line with the proposed RDoC framework, our results suggest cool EF tasks elicit top-down cognitive control [72] and are differentiated from positive/negative valence processes which involve a more complex network of brain regions [73].

Our results demonstrate that it is difficult to disentangle specific positive and negative valence constructs within some decision-making tasks. In contrast to the ‘hungry donkey’ task, where reward-seeking (positive valence) is separated from loss sensitivity (negative valence), the positive and negative valence measures extracted from the BELT decision-making task were combined into a single factor. We, therefore, created a combined positive/negative valence construct reflecting impulsive behaviour driven by heightened emotions irrespective of valence (i.e., emotional impulsivity) [69]. Previous research shows that the tendency towards regrettable behaviour in states of high emotion predicts a vast range of severe internalising and externalising problems [74, 75], and thus may be an important construct for further investigation.

We found that ADHD symptoms were associated with poor cognitive control, whereas anxiety was associated with better cognitive control, which aligns with previous research demonstrating that co-occurring anxiety may reduce some cognitive problems in children with clinical symptoms [26, 66, 76]. Our finding that only ADHD was associated with poorer cognitive control fits with theories of self-control difficulties under ‘cool’ (non-emotional) contexts in ADHD, whereas problems executing self-control in ‘hot’ emotional contexts is associated with disruptive behaviour and emotional disorders [9, 11, 18, 77].

We found that low and high reward-seeking predicted variance in depression and ODD symptoms, respectively. Previous research indicates that ODD and depression are associated with blunted responsiveness to reward but result in different behaviours: whilst depression is associated with risk aversion [12], ODD is associated with high reward-seeking [78]. Because ODD and depression were not independently associated with emotional impulsivity, which reflects sensitivity to both reward and loss, the results suggest that ODD and depression are specifically associated with dysfunctional positive valence processes, as opposed more a general mechanism of impulsivity which spans multiple RDoC domains. Where associations between general impulsivity and ODD or depression have been found [79], this may be because co-occurring symptoms (e.g., depression in ODD) were not accounted for.

We found that emotional impulsivity was not independently associated with any disorder symptom scores. Using the same BELT task, Humphreys and Lee (2011) [80] found that children with comorbid ADHD and ODD demonstrated both more reward-seeking and greater loss sensitivity than single disorder groups and controls. We may have found no associations between ADHD or ODD and emotional impulsivity because we examined independent associations rather than the combined effects of these disorders. Children with both ADHD and ODD may react more strongly to negative events because of poor cognitive and affective regulation [11, 18, 78]. We found that children with moderate-to-high problems performed more poorly on the BELT task than children without problems; emotional impulsivity may, thus, be indicative of a broader or more general dysregulation syndrome than a disorder-specific problem.

The construct corresponding to negative valence processes was significantly inversely correlated with ADHD, ODD and depression symptoms. However, after controlling for co-occurring symptoms, no associations between symptom scores and negative valence remained significant, suggesting that loss sensitivity was not independently associated with specific symptoms. Loss sensitivity could be associated with irritability [81], a phenotype implicated in ADHD, ODD and depression [82]. Controlling for comorbidity will dampen associations because of shared variance and reductions in statistical power. Irritability and loss sensitivity in young children could be another transdiagnostic process associated with different clinical symptom dimensions [83, 84].

## Strengths and limitations

The current study is one of the first to examine positive and negative valence processes using lab-based cognitive tasks in a relatively large sample of young, pre-diagnosed children. Children were recruited from the community via educational professionals; our sample is, therefore, more representative of those exhibiting self-regulation problems at school than a volunteer sample recruited via parents.

For the examination of cool EF, we were able to compare our sample to typically developing children using norm scores, but there were no norm referenced scores for the decision-making tasks (BELT, Hungry Donkey). We, therefore, used children who had been referred by their teachers but whose teacher SDQ scores were in the ‘close to average’ range. This may explain why an elevated proportion of children had below-average performance on some cognitive tasks (e.g., inhibition, decision-making). However, this comparison group is less likely to differ on some confounding factors (e.g. socioeconomic status, education) which can exaggerate differences between at-risk samples and controls.

The current study also had several limitations. First, caution should be taken when considering the results of our factor analysis, because the number of variables used was small relative to the number of factors extracted. The results of our factor analysis also highlight that separation of positive and negative valence processes is difficult to achieve, because in most decision-making tasks, reward and loss sensitivity processes interact and do not operate independently. Second, because this paper focussed on associations between multiple dimensions of self-regulation and clinical symptoms, the results should be considered exploratory owing to the fairly large number of analyses carried out. Third, a correlational design was used; thus, we cannot infer the direction of causality between self-regulation processes and clinical symptoms. Fourth, although performance on decision-making tasks has been shown to elicit autonomic responses and to correspond with real-life risk-taking behaviour, such as substance use, gambling and criminal behaviour [53, 62], it may be that the gains and penalties within the game do not activate motivational processes to the same intensity as when the child has access to actual (e.g., monetary) gains or losses. Therefore, our results require replication using tasks with greater ecologically validity (e.g., Schoorl et al. [86]).

Finally, in line with other research, our sample had a higher proportion of boys than girls with moderate-to-high dysregulation symptoms [85, 87]. However, we found no sex differences on our measures of cognitive control and decision-making (except for response inhibition, where girls performed more poorly) indicating that being a girl is not entirely protective against difficulties in specific self-regulation processes. Emotion regulation difficulties in girls are less noticed by educational professionals [88]; thus, further research in larger samples of girls exploring the cognitive processes associated with symptoms of anxiety and depression is needed to ensure that girls with emerging emotional problems are not overlooked.

### Implications and future directions

Because the current study used a correlational design and we, therefore, cannot infer causality, there is a need for longitudinal research to verify the causal links between SR processes and clinical symptoms. Surface-level manifestations of clinical symptoms can change over time (e.g., ODD can evolve into depression) [89, 90], whereas SR problems may be more stable and consistently mediate links between early genetic and environmental factors and later adverse outcomes [77]. Instead of relying on disorder classification systems, assessing SR in clinical or educational settings may be useful, not only to understand underlying psychological difficulties but also to optimise the delivery of personalised interventions [91, 92].

We found that negative valence processes and emotional impulsivity were not independently associated with clinical symptoms, highlighting that some RDoC processes may reflect general rather than disorder-specific problems. Other studies exploring the factor structure of cognitive, emotional and behavioural regulation difficulties in children have found that both specific and general factors contribute to clinical symptoms [15]. Further research using both specific and broad measures of dysregulation symptoms, such as the CBCL-Dysregulation Profile [93], is needed to establish the processes which are disorder specific, and those which are implicated more generally across psychopathology. If it is found that sensitivity to loss or emotional impulsivity are general risk factors, they may be useful targets for general prevention interventions [94].

## Conclusion

Self-regulation (SR) processes mediate the link between early familial influences and adverse developmental outcomes [77]. We found that young school-referred children with varying cognitive, emotional and behavioural problems exhibit difficulties with cognitive control and decision-making. ADHD and disruptive behaviour symptoms were independently associated with poor cognitive and motivational control, respectively, whereas emotional disorder symptoms were associated with relative strengths on tasks assessing cognitive control (anxiety) and risk-adverse decision-making (depression). We also found that the separation of positive and negative valence processes is complex and hard to achieve, with some evidence of transdiagnostic dysregulation across disorders. Further research using the RDoC framework and dimensional approaches will ultimately offer us better opportunities for intervention, not only targeting specific disorder symptoms, but also more general functional difficulties that span multiple diagnoses.

### Supplementary Information

Below is the link to the electronic supplementary material.

Supplementary file1 (DOCX 27 KB)

## Data Availability

The conditions of our ethics approval do not permit public archiving of anonymised study data. Readers seeking access to the data should contact the lead investigator (SHMvG) or the local ethics committee at the School of Psychology, Cardiff University. Access will be granted to named individuals in accordance with ethical procedures governing the reuse of sensitive data. Specifically, requestors must complete a formal data sharing agreement with the lead investigator.

## Footnotes

1 Because Levene’s test for equality of variances was significant (*p* < 0.05), *t* test result reflects analyses where equal variances were not assumed.
