# Supplementary material for: Cool and hot executive function problems in young children: linking self-regulation processes to emerging clinical symptoms
Source: Eur Child Adolesc Psychiatry. 2024 Jan 6;33(8):2705–18. doi: 10.1007/s00787-023-02344-z (PMC11272683; doi:10.1007/s00787-023-02344-z)
Supplement: Supplementary file 1 — Supplementary file1 (DOCX 27 KB) [file 787_2023_2344_MOESM1_ESM.docx]

Supplementary Table 1

*Multiple regression analyses examining formed RDoC constructs as predictors of ADHD symptom severity, controlling for co-occurring symptoms age, sex and verbal ability*

| Formed RDoC constructs | Statistic | Symptom dimensions |
| --- | --- | --- |
| Step 1: | ∆R^2^ | .02 |
| Age | ß  95% CI | .08  [-.06, .20] |
| Sex | ß  95% CI | .12  [-.70, 5.98] |
| Verbal ability | ß  95% CI | -.02  [-.12, .09] |
| Step 2 | ∆R^2^ | .31** |
| Anxiety | ß  95% CI | .03  [-.11, .17] |
| Depression | ß  95% CI | .06  [-.11, .22] |
| ODD | ß  95% CI | .51**  [.41, .74] |
| Step 3 | ∆R^2^ | .05** |
| Low reward-seeking (+ valence) | ß  95% CI | .016  [-1.13, 1.45] |
| High cognitive control (cognitive systems) | ß  95% CI | -.20**  [-3.28, -.73] |
| High emotional impulsivity (+ / - valence) | ß  95% CI | -.02  [-1.50, 1.03] |
| High loss sensitivity (- valence) | ß  95% CI | -.12  [-2.54, .03] |

*Note.* ODD = Oppositional Defiance Disorder. * = p <.05, ** = p<.1

Supplementary Table 2.

*Multiple regression analyses examining formed RDoC constructs as predictors of anxiety symptom severity, controlling for co-occurring symptoms age, sex and verbal ability*

| Formed RDoC constructs | Statistic | Symptom dimensions |
| --- | --- | --- |
| Step 1: | ∆R^2^ | .01 |
| Age | ß  95% CI | .03  [-.11, .17] |
| Sex | ß  95% CI | -.05  [-5.02, .07] |
| Verbal Ability | ß  95% CI | -.06  [-.16, .07] |
| Step 2 | ∆R^2^ | .38** |
| ADHD | ß  95% CI | .03  [-.12, .06] |
| Depression | ß  95% CI | .58*  [.46, .75] |
| ODD | ß  95% CI | .04  [-.14, .25] |
| Step 3 | ∆R^2^ | .40 |
| Low reward-seeking (+ valence) | ß  95% CI | .02  [-1.13, 1.63] |
| High cognitive control (cognitive systems) | ß  95% CI | .13*  [.06, 2.83] |
| High emotional impulsivity (+ / - valence) | ß  95% CI | .01  [-1.27, 1.43] |
| High loss sensitivity (- valence) | ß  95% CI | .04  [-.90, 1.88] |

*Note.* ADHD = Attention Deficit Hyperactivity Disorder. ODD = Oppositional Defiance Disorder. * = *p* <. 05, ** = *p* <. 01

Supplementary Table 3.

*Multiple regression analyses examining formed RDoC constructs as predictors of depression symptom severity, controlling for co-occurring symptoms age, sex and verbal ability*

| Formed RDoC constructs | Statistic | Symptom dimensions |
| --- | --- | --- |
| Step 1: | ∆R^2^ | <.001 |
| Age | ß  95% CI | -.01  [-.15, .09] |
| Sex | ß  95% CI | -.02  [-3.98, 3.05] |
| Verbal Ability | ß  95% CI | -.03  [-.13, .09] |
| Step 2 | ∆R^2^ | .49** |
| Anxiety | ß  95% CI | .48**  [.35, .56] |
| ADHD | ß  95% CI | .05  [-.09, .18] |
| ODD | ß  95% CI | .34  [.35, .57] |
| Step 3 | ∆R^2^ | .02 |
| Low reward-seeking (+ valence) | ß  95% CI | .13*  [.20, 2.54] |
| High cognitive control (cognitive systems) | ß  95% CI | -.01  [-1.27, 1.15] |
| High emotional impulsivity (+ / - valence) | ß  95% CI | .02  [-.92, 1.41] |
| High loss sensitivity (- valence) | ß  95% CI | -.08  -2.08, .30] |

*Note.* ADHD = Attention Deficit Hyperactivity Disorder. ODD = Oppositional Defiance Disorder. * = *p* <. 05, ** = *p* <. 01

Supplementary Table 4.

*Multiple regression analyses examining formed RDoC constructs as predictors of ODD symptom severity, controlling for co-occurring symptoms age, sex and verbal ability*

| Formed RDoC constructs | Statistic | Symptom dimensions |
| --- | --- | --- |
| Step 1: | ∆R^2^ | .01 |
| Age | ß  95% CI | -.02  [-.13, .10] |
| Sex | ß  95% CI | -.05  [-3.84, 2.05] |
| Verbal Ability | ß  95% CI | -.05  [-.12, .06] |
| Step 2 | ∆R^2^ | .44** |
| Anxiety | ß  95% CI | .04  []-.08, .14] |
| ADHD | ß  95% CI | .42**  [.26, .47] |
| Depression | ß  95% CI | .37**  [.19, .43] |
| Step 3 | ∆R^2^ | .02 |
| Low reward-seeking (+ valence) | ß  95% CI | -.13*  [-2.23, -.18] |
| High cognitive control (cognitive systems) | ß  95% CI | .09  [-.26, 1.83] |
| High emotional impulsivity (+ / - valence) | ß  95% CI | .01  [-.92, 1.12] |
| High loss sensitivity (- valence) | ß  95% CI | -.01  [-1.10, .99] |

*Note.* ADHD = Attention Deficit Hyperactivity Disorder. * = *p* <. 05, ** = *p* <. 01
